# “Hollow Pursuits” and the Right Not to Be Simulated: privacy, autonomy, and the holodeck

**DOI:** 10.3389/fgene.2026.1984132

**Published:** 2026-10-30

**Authors:** Gareth W. Young

**Affiliations:** School of Computer Science and Statistics, Trinity College Dublin, Dublin, Ireland

**Keywords:** artificial intelligence, digital humans, extended reality, holodeck, privacy, simulation, Star Trek: The Next Generation

## Abstract

Emerging artificial intelligence and extended reality technologies increasingly enable the construction of interactive digital representations of identifiable people from their faces, voices, behavioral traits, personal data, and other records. Such systems raise a problem that extends beyond conventional questions of informational privacy or synthetic likeness. Drawing on the Holodeck in Star Trek: The Next Generation, particularly “Hollow Pursuits,” “Booby Trap,” and “Galaxy’s Child,” this paper examines this problem through the lens of relational access. While surveillance provides epistemic access to a person and profiling provides inferential access, person-specific simulation can provide relational access to an interactive surrogate capable of supporting social interactions associated with the represented person. The surrogate enables person-specific interaction in the represented individual’s absence, creating a form of radically non-reciprocal sociality in which the individual cannot determine whether or how their identity is made interactively available. Building on work on privacy, autonomy, synthetic likeness, and digital replicas, the paper proposes a Right Not to Be Simulated: a presumptive autonomy and authority claim against the unauthorized creation or use of person-specific interactive surrogates.

## 1 Introduction

What happens when a person can be present without physically being there? *Star Trek: The Next Generation* (TNG), which follows the crew of the starship USS *Enterprise-D* in the 24th century, repeatedly explores this question through the Holodeck. The Holodeck is used by the crew for recreation, training, and other simulated experiences; it is a fictional technology capable of generating perceptually convincing, interactive environments populated by responsive characters. Within these simulations, representations of real individuals can speak, converse, form relationships, and participate in situations that their real-world counterparts have neither experienced nor authorized. The resulting ethical problem is not simply that information about a person has been collected or revealed. Rather, that information has become sufficiently available to produce an interactive artificial version of them.

For much of the Holodeck’s fictional history, this problem could be treated as purely speculative. However, recent advances in artificial intelligence (AI), digital humans, and immersive XR technologies have made increasingly convincing digital replicas of identifiable people possible, including representations based on their faces, voices, performances, psychological characteristics, and behavioral traces (Öhman, 2022; Rothman, 2025; Karpus and Strasser, 2025; Bosher, 2026). Work on AI clones and Real Persona Social AI (RPSAI) similarly shows how person-specific data can be transformed into interactive generative likenesses capable of producing new speech and behavior in an individual’s absence (Lee et al., 2023; Loewith, 2026). Contemporary systems remain far from the Holodeck’s fictional capabilities, but they make the underlying problem increasingly concrete: the trajectory is toward person-specific computational models that can be questioned, manipulated, embodied, and placed in simulated social situations.

One indication of this trajectory appears in Park et al.’s recent work on person-specific generative agents. Drawing on qualitative interviews with 1,052 participants, they built generative agents intended to represent individual participants and evaluated their ability to reproduce aspects of those participants’ attitudes and behavior (Park et al., 2024). Although the study is a preprint and far removed from the embodied and socially sophisticated simulations imagined by the Holodeck, it demonstrates that information gathered about an identifiable person today can already be used to construct a generative model designed to respond in ways associated with that individual.

Today’s privacy researchers offer important insights into aspects of this problem. Privacy scholarship has examined surveillance, information collection, profiling, data aggregation, and how information processing can construct inferential knowledge about individuals (Nissenbaum, 2004; 2010; Cohen, 2012). Nissenbaum’s theory of contextual integrity, in particular, moves privacy beyond secrecy or individual control by asking whether information flows are appropriate to the social context in which they occur (Nissenbaum, 2004; 2010). Cohen similarly treats information practices as participating in the formation of subjectivity rather than merely recording facts about an individual (Cohen, 2012). These approaches remain fundamental, but *Star Trek*’s Holodeck simulations pose additional challenges.

Surveillance provides epistemic access to a person by allowing others to observe them. Profiling provides inferential access, producing claims about what a person is like or what they might do. Person-specific simulation can create a different possibility, which I call relational access: access to an interactive, person-specific surrogate capable of supporting forms of social interaction ordinarily associated with the represented person. Relational access does not mean that the represented person is literally accessed through the simulation, nor that they participate in the resulting encounter. The person remains absent. What becomes available is a computational surrogate that can speak, respond, and interact in ways associated with them. Surveillance, profiling, and simulation may overlap or provide inputs to one another, but they need not occur sequentially. Figure 1 distinguishes the forms of access each can afford.

**FIGURE 1:**
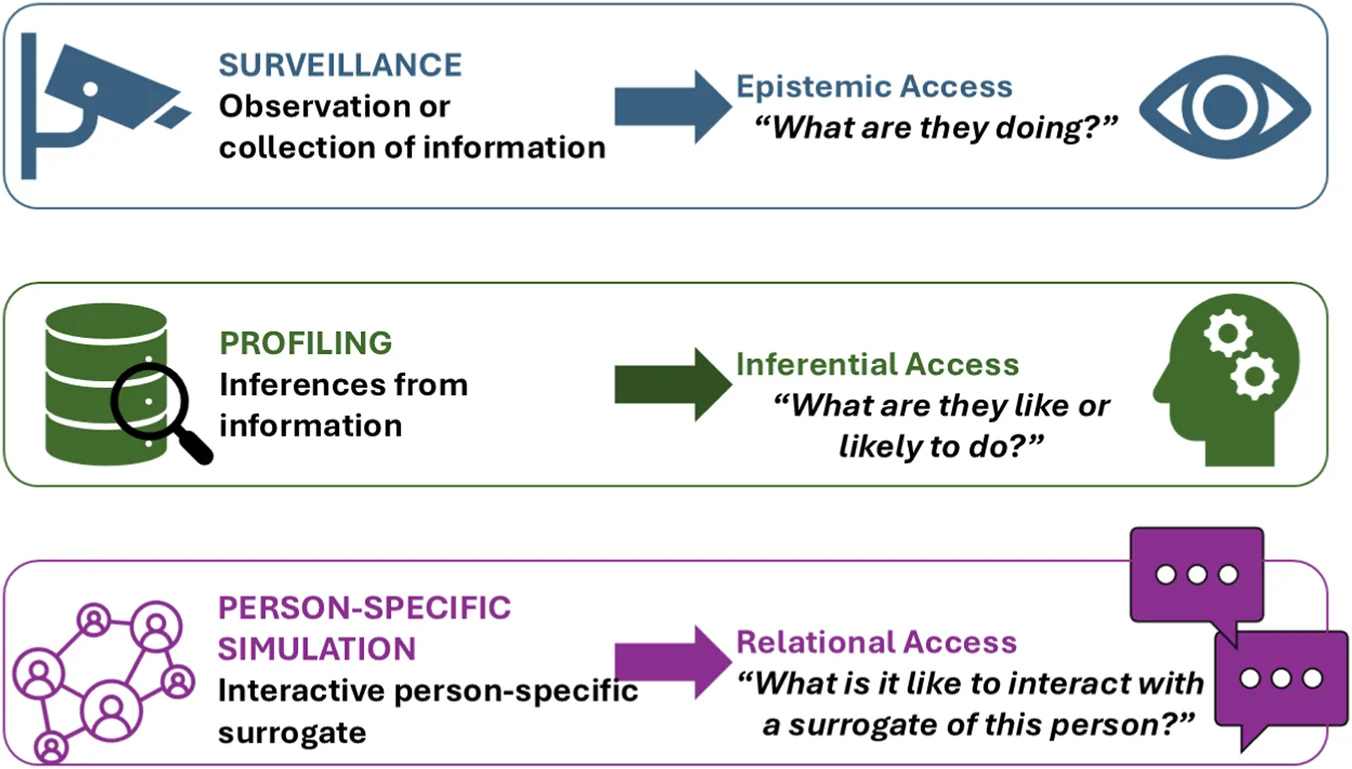
Epistemic, inferential, and relational forms of access. These categories are analytically distinct rather than stages in a necessary sequence. Person-specific simulation may draw upon observed, provided, or inferred information, but relational access refers specifically to interaction with a person-specific surrogate.

The Holodeck makes distinctions among these types of access unusually salient. Its simulations expose a recurring gap between possessing information about someone and having permission to use that information to construct an interactive surrogate associated with them. This gap is increasingly visible outside of science fiction. Recent work on deepfakes and digital replicas has questioned whether individuals possess moral or legal claims over synthetic representations of their faces, voices, likenesses, and identities (Chesney and Citron, 2019; Öhman, 2022; Rothman, 2025; Bosher, 2026). Deepfakes have already raised concerns about privacy, reputation, deception, and the broader social consequences of synthetic representations of identifiable people (Chesney and Citron, 2019). Karpus and Strasser (2025) go further, considering digital replicas as possible extensions of personhood and asking whether individuals may possess privileged claims over both their creation and the personal data from which they are produced.

Existing work increasingly recognizes that person-specific AI can transform personal data into generative likenesses, undermine social authorship, and conscript an individual’s identity into unauthorized synthetic performances (Loewith, 2026; Kirkpatrick, 2026). This establishes that synthetic replication can implicate interests beyond conventional informational privacy. This paper builds on these accounts by focusing on what becomes possible when the resulting representation is interactive. Once a computational surrogate becomes socially responsive, it does not merely speak or act in ways associated with the represented person; another party can question it, respond to it, encounter it repeatedly, and potentially develop a history of interaction with it.

The person creating or using a simulation can interact with, modify, and act upon the surrogate. The represented individual is not a participant in that simulated encounter, yet their identity provides the surrogate’s person-specific basis. They may not know that the encounter has occurred and have no opportunity to determine whether or how their identity is used within it. This creates a form of radically non-reciprocal sociality in which one party gains access to person-specific interaction through a surrogate, while the represented individual remains absent from the interaction and lacks corresponding agency over its creation or terms.

The Holodeck has long served as a cultural model for thinking about computationally generated and participatory worlds, most notably in Murray’s account of digital narrative and agency (Murray, 1997). Here, however, it functions as a device for anticipatory governance. Anticipatory governance focuses on building capacities to consider and respond to the social consequences of emerging science and technology before those consequences become settled (Guston, 2014). This approach builds on work using fictional and interactive media to make emerging questions of AI ethics, surveillance, and data politics available for critical examination (Young et al., 2025). The fictional technologies considered here are treated not as predictions, but as conceptual cases through which emerging ethical and governance problems can be examined. Science fiction is useful in this context not because it reliably predicts technological development, but because it can make possible technological arrangements and their ethical tensions available for examination.

The three *TNG* episodes examined here are treated as conceptual cases rather than as a systematically sampled corpus. “Booby Trap” examines the construction of a surrogate from professional information, “Hollow Pursuits” its private creation and use, and “Galaxy’s Child” the confrontation between surrogate-mediated expectations and the represented person. Other episodes across the franchise raise related questions, including *Star Trek: Deep Space Nine*’s “Meridian” and *Star Trek: Voyager*’s “Author, Author,” but the aim here is not a franchise-wide survey.

Against this backdrop, this paper develops the concept of a Right Not to Be Simulated: a presumptive normative claim against the unauthorized creation or use of sufficiently person-specific interactive surrogates. The proposal is not an absolute prohibition on depiction, modeling, fictionalization, impersonation, or simulation. Nor does it assume that interacting with a surrogate amounts to interacting with the represented person. Instead, it asks whether individuals possess an autonomy or authority interest in whether their identity may be used to create a socially responsive surrogate that others can use to conduct person-specific interactions in their absence. Therefore, the argument proceeds from the distinction among information about a person, representations of that person, and the interactive availability of a surrogate constructed in relation to that person.

## 2 The holodeck as a technology of simulation

The Holodeck occupies a unique place within *Star Trek*’s technological imaginings. Aboard the *Enterprise-D*, it allows crew members to enter computer-generated environments for recreation, training, and other purposes, with simulated surroundings and characters that respond dynamically to their actions. It is presented as a computational system capable of constructing perceptually convincing, socially responsive worlds in which physical space, synthetic objects, narrative structures, and simulated people can be experienced as if they were immediately present to the immersant. Users enter these simulations, move through them, speak with their inhabitants, manipulate their environments, and participate in unfolding situations. In contemporary terms, the Holodeck anticipates a convergence of XR, AI, embodied interaction, and computational agents, in which the boundaries between environment, interface, and simulated social actors become increasingly difficult to distinguish (Young and Dawkins, 2023).

This matters because the Holodeck does more than accurately reproduce appearances. Its simulations of people are interactive and generative. A representation of a person can respond to questions, remember prior exchanges, display apparently coherent motivations, and adjust its behavior based on what the user says or does. Such characters function less like a recording or conventional digital likeness and more like a computationally intelligent social surrogate. The system does not merely reproduce what a person looks or sounds like; it can generate a person-specific surrogate with which another individual can sustain an encounter. Contemporary XR research already treats multimodal sensory feedback, embodiment, presence, and increasingly naturalistic interaction as central dimensions of immersive experience (Young and Dawkins, 2023).

*Star Trek: The Next Generation* repeatedly shows that such simulations can be derived from information about identifiable people. Holodeck characters can be based on historical figures, fictional characters, crew members, or on combinations of existing records and computational inference. The boundary between reconstruction and invention is consequently blurred. A simulation may begin with recorded information about a person but extend beyond it by generating speech, actions, preferences, emotional responses, or hypothetical behavior the original person never expressed. In this sense, the Holodeck creates an intelligent model associated with a person–one capable of generating novel behavior under circumstances that may never have occurred in that person’s life.

In “Hollow Pursuits,” Enterprise engineer Reginald Barclay retreats into Holodeck fantasies featuring exaggerated versions of several colleagues, including Counselor Deanna Troi, Commander William Riker, Wesley Crusher, and Geordi La Forge (Caves, 1990). In these scenarios, Barclay can reshape their personalities, appearances, roles, and relationships with him. The simulations range from comic and heroic fantasies to more intimate representations, most notably a sexualized version of Troi.

Importantly, the episode does not settle the morality of Barclay’s private fantasies. When members of the crew discover the program, their reactions combine embarrassment, anger, concern about Barclay’s behavior, and uncertainty about the limits that should apply to private Holodeck use. This ambiguity matters. Imagining a colleague, even within a private fantasy, is not equivalent to computationally instantiating that colleague as a responsive character. The latter externalizes the fantasy by instantiating the colleague as an interactive artifact whose appearance and behavior can be generated and manipulated. Therefore, “Hollow Pursuits” provides a useful boundary case for asking what, if anything, changes when private imagination becomes a person-specific interactive simulation.

The Leah Brahms storyline approaches person-specific simulation from a different angle. In “Booby Trap,” La Forge consults technical records associated with Dr. Leah Brahms and asks the *Enterprise* computer to create a Holodeck representation of her to help solve an engineering problem (Roman et al., 1989). The initial purpose is professional and instrumental: La Forge seeks an interactive way to work with Brahms’s technical knowledge. As the simulation develops, however, the encounter is shaped jointly by La Forge’s requests and choices and by the computer’s generative behavior. La Forge invites the system to develop the representation beyond a straightforward retrieval of Brahms’s technical knowledge, while the computer incorporates additional information about Brahms and generates conversational and affective responses. The resulting interaction moves beyond technical consultation, and La Forge becomes emotionally attracted to the simulated Brahms. The resulting character does not merely retrieve statements Brahms previously made; it generates novel responses in her likeness that the real Brahms neither chose nor authorized.

The distinction becomes explicit in “Galaxy’s Child,” when the real Leah Brahms later visits the *Enterprise* and meets La Forge in person (Hurley, 1991). La Forge approaches her with expectations shaped by his earlier experience with the Holodeck surrogate, only to find that the real Brahms does not behave as the simulation did. Brahms subsequently discovers the program and reacts angrily to what she sees. The importance of this encounter is not that Brahms unknowingly participated in La Forge’s earlier interaction; she had not. Rather, La Forge had accumulated an interactional history with a person-specific surrogate associated with Brahms, and that history subsequently affected his encounter with the real person. The simulation looks like Brahms, speaks in her voice, draws on her expertise, and generates social behavior associated with her identity, even though that behavior is partly produced through computational inference.

This feature of the Holodeck can be understood through the distinction between representation and simulation. Baudrillard’s account of simulation and simulacra is useful here for distinguishing simulation from straightforward representation (Baudrillard, 1994). Once operational, a person-specific simulation can generate behaviors, responses, and meanings that cannot be straightforwardly attributed to the individual from whom the simulation was derived. The simulated Leah Brahms remains recognizable because of her relationship to the real Brahms, but the social encounter La Forge experiences is generated in part by the simulation itself. Therefore, the ethical problem cannot be reduced to whether the surrogate accurately predicts what Brahms would say or do. An inaccurate simulation may still trade on the identity of a real person, while a highly convincing one may create a stronger impression of interacting with that person’s likeness or personality.

The Holodeck differs from conventional 2D media by collapsing the distance between representation and participation. Contemporary volumetric video already complicates the distinction between recorded representation and spatial presence by rendering captured performances of real people as three-dimensional media within interactive XR environments (Young et al., 2023). Yet such representations remain fundamentally recorded performances. A photograph, film, or written description presents a person for observation but does not ordinarily respond to the observer. This distinction is increasingly visible in contemporary social AI. Loewith (2026) distinguishes interactive RPSAI from conventional deepfakes precisely because such systems do not merely present fixed synthetic representations but can engage in fluid, context-sensitive interaction while performing the social likeness of an identifiable person.

The difference is not simply one of fidelity but of the kind of access the representation affords. A sufficiently sophisticated person-specific simulation makes an interactive surrogate available, not the represented person themselves. The user can ask questions, test reactions, alter circumstances, repeat encounters, modify the simulated person’s appearance or behavior, and construct situations aligned with their own interests or desires. The represented individual need not know that any of this has occurred. Nor are they literally a participant in what occurs. The asymmetry instead arises because one party can conduct person-specific interactions through a surrogate whose identity and intelligibility depend on another person, while that represented person remains absent from the creation and conduct of those interactions.

This asymmetry becomes clearer when simulation is compared with adjacent informational practices. As noted, surveillance provides epistemic access to a person by making them observable. Profiling provides inferential access by enabling claims about their attributes, preferences, or likely behavior to be derived from available information. Neither practice necessarily produces an interactive surrogate, and person-specific simulation need not arise from either in a fixed sequence. Simulation can instead provide a distinct affordance: relational access, understood here as access to an interactive, person-specific surrogate capable of supporting social interactions associated with the represented person. The resulting simulation may be questioned, befriended, manipulated, sexualized, insulted, experimented upon, or placed in imagined relationships, regardless of whether the represented individual would consent to their identity being used as the basis for such interactions.

The Holodeck’s significance for the argument presented lies in its ability to transform information about an identifiable person into a socially responsive surrogate. Once such a representation can act, respond, remember, and generate novel behavior, the question is no longer simply one of informational accuracy or visual likeness, but rather who may create and interact with it and on what terms.

## 3 When simulation becomes a privacy problem

The privacy problem posed by person-specific simulation begins with the collection and use of personal information but does not end there. Conventional accounts of privacy distinguish harms arising from information collection, processing, dissemination, and misuse, including surveillance, aggregation, identification, secondary use, appropriation, distortion, and intrusion (Solove, 2005). These concerns apply directly to simulation technologies. Constructing a convincing model of a real person may draw on photographs, recordings, behavioral traces, biometric information, biographical details, medical information and, potentially, genetic data, many of which may already be available in digital systems. Whether those materials were obtained lawfully, whether their subsequent processing is compatible with the purposes for which they were collected, and whether resulting information flows are appropriate to their social context are familiar privacy and data-protection questions (Nissenbaum, 2004; 2010). Person-specific simulation adds a further question: whether information that may legitimately be held or processed for one purpose may be assembled into an interactive surrogate capable of supporting person-specific social interaction.

The Leah Brahms case offers a useful way to distinguish what existing privacy and autonomy accounts already explain from what relational access is intended to add. In “Booby Trap,” the Holodeck reconstructs Brahms from technical records and other available information to help Geordi La Forge with an engineering problem. In that context, access to her professional work appears legitimate. However, the simulation moves beyond its instrumental purpose. La Forge converses with the holographic Brahms and develops an emotional attachment to a version of her that responds to him in ways the real Brahms neither chose nor authorized. When Brahms later discovers the simulation in “Galaxy’s Child,” her reaction exposes a gap between informational and interpersonal permissions. Contemporary RPSAI illustrates a comparable technical possibility, converting person-specific data from informational input into a generative likeness capable of autonomous social interaction (Loewith, 2026). Therefore, the case is not difficult because existing privacy theories have nothing to say about it. On the contrary, several established accounts explain important parts of what has occurred.

Nissenbaum’s account of contextual integrity asks whether information flows align with norms appropriate to a given social context (Nissenbaum, 2004; 2010). This already explains a substantial part of the Brahms case. Her professional information is available to La Forge because he is attempting to solve an engineering problem aboard the *Enterprise*. The difficulty arises when information made available within that professional context becomes input to a socially responsive representation that then operates within a more personal and intimate encounter. Information appropriate to an engineering setting thereby acquires a social function different from the one that initially justified its availability.

Lawful or consensual access to personal information for one purpose does not determine whether every subsequent transformation is permissible. A photograph may be lawfully obtained without authorizing its animation, just as professional records may be available without authorizing their use to build a conversational model. Therefore, person-specific simulation makes familiar questions of purpose limitation and subsequent processing particularly acute because stored information can become a new interactive capability. In the Brahms case, permission to use information associated with her professional expertise does not, by itself, settle whether that information may be used to construct a surrogate for personal interaction. Permission to know is not permission to instantiate.

Privacy scholarship also offers reasons to resist treating the Brahms case solely as a question of secrecy or control over isolated pieces of information. Rachels argues that privacy is important, in part, because control over access to ourselves helps structure different kinds of social relationships (Rachels, 1975). Floridi similarly links informational privacy to personal identity, treating interference with a person’s informational constitution as potentially affecting the person rather than merely something they possess (Floridi, 2005). These accounts help explain why transforming person-specific information into a socially responsive surrogate can matter even when the underlying information is neither secret nor unlawfully obtained.

Existing work on synthetic likeness, personality rights, digital replicas, and avatar proxies explains another aspect of the problem (Mashinini, 2020; Sweeney, 2023; Rothman, 2025; Bosher, 2026). These approaches focus on related questions concerning how a person’s face, voice, identity, or likeness is used and how conduct may be attributed to them. Sweeney’s account of avatars as proxies is particularly relevant to the possibility that an avatar may stand for and act in place of a represented person, raising questions about when avatar-generated conduct may be treated as theirs (Sweeney, 2023). More broadly, these concerns point toward an interest in representational autonomy: a person may object to a representation that distorts their identity or attributes unwanted conduct to them, even when the underlying information was lawfully obtained. This concern is especially clear when a simulation generates novel behavior. The holographic Brahms does not merely reproduce an existing recording; it says and does things the real Brahms did not say or do. Therefore, a person may have a representational interest in whether such generated conduct is presented through a model recognizably associated with them, regardless of whether another person subsequently forms any attachment to it.

This point is also consistent with arguments that the wrong involved in deepfakes need not be exhausted by deception or demonstrable downstream harm. De Ruiter’s account of deepfakes is useful because it directs attention to what may be objectionable about unauthorized synthetic representation itself (de Ruiter, 2021). Person-specific simulation inherits these representational concerns, but interactivity raises an additional question about what others may do *with* the resulting representation.

This creates a further distinction between control over a representation and control over the relations that representation makes possible. A person may have an interest in whether a synthetic likeness of them is created, whether it is accurate, and how it is distributed. Simulation adds the question of whether that representation may be made socially responsive and available for interpersonal use. In this sense, representational autonomy and relational autonomy overlap but are not identical. The former concerns how a person is presented through identity-linked representations; the latter concerns the person’s capacity to negotiate the social relations mediated by that identity. A simulation can interfere with both at once by producing a version of the person that speaks, responds, and participates in encounters while remaining beyond their control.

Kirkpatrick’s authority-interest account offers further insight into the Brahms case: algorithmic simulation may usurp a person’s authority over the use of their identity as a generative resource (Kirkpatrick, 2026). This helps explain why unauthorized synthetic performance can be wrongful. It also helps distinguish two questions that need not be identical: who has authority to create or generate conduct using a person’s identity, and what forms of interaction may subsequently occur with the resulting surrogate. The first concerns authority over synthetic performance, while the second becomes especially salient once that performance is interactive.

Taken together, these accounts explain much of what is troubling about the Brahms case. Contextual integrity focuses on the movement of person-specific information across social purposes and contexts. Data-protection principles raise questions about purpose, compatibility, and subsequent processing. Likeness and digital-replica protections address uses of identity-linked features. Representational autonomy addresses Brahms’s interest in how a synthetic version of her is presented, while authority-based accounts address the use of her identity as a generative resource. Relational access is not proposed as a replacement for these explanations. It identifies an additional affordance that arises when these materials are assembled into a socially responsive surrogate: another person gains the capacity for person-specific interaction with that surrogate in the represented individual’s absence. Repeated interaction may allow the user to accumulate an interactional history with the surrogate, as occurs in the Brahms case, but such a history is one possible consequence of relational access rather than a necessary condition for it.

That interactional history is not literally a relationship with Brahms. She is not present in it, does not participate in it, and is not responsible for what the surrogate says or does. Nevertheless, La Forge can converse with the model, respond emotionally to it, develop expectations through repeated interaction, and carry those expectations into his encounter with the real Brahms. “Galaxy’s Child” makes this particular consequence visible precisely because the real Brahms differs from the surrogate with which La Forge has already interacted. Therefore, the distinctive question raised by relational access is not whether a simulation somehow provides access to the person themselves, but whether another party should be entitled to instantiate or use a person-specific surrogate for social interaction in that person’s absence. Repeated interaction, and the interactional histories it can produce, may strengthen this concern without defining it.

This distinction aligns with HCI research on AI clones, which identifies a tripartite relationship among the user, the source individual, and the clone and finds that interactions with a surrogate may affect the relationship between the user and the person represented (Lee et al., 2023). Relational access names the specific capability available to the user within this arrangement: access to interaction with the surrogate. Its ethical importance may stem in part from effects on the source person or their relationships, but, as the following section argues, the normative claim need not depend entirely on such downstream consequences.

The resulting privacy problem is, therefore, neither entirely new nor fully reducible to any single existing account. Person-specific simulation can implicate informational privacy, contextual integrity, identity, likeness, representational autonomy, and authority simultaneously. Relational access underscores the importance of making the resulting surrogate available for interaction. The remaining question is normative: whether the represented person has a presumptive claim against that form of interactive availability, even when the simulation causes no demonstrable further harm. It is this question that motivates the proposed Right Not to Be Simulated.

## 4 A Right Not to Be Simulated

If person-specific simulation creates a form of relational access that conventional accounts of data privacy do not exhaust, a further normative question follows: should individuals have a claim over whether interactive surrogates associated with them may be made available to others? I propose that this concern can be expressed as a Right Not to Be Simulated. The right is proposed here as a moral claim about what individuals should be entitled to control, rather than as an assertion that an equivalent freestanding legal right presently exists. Such a right would not amount to absolute ownership over every representation, description, or computational model associated with a person. Rather, it would establish a presumptive claim against the creation or use of an interactive, person-specific simulation capable of standing in for an identifiable individual without that individual’s knowledge or authorization. Its purpose would be to protect not only information about a person but also their authority over whether their identity is used as the basis for a socially responsive surrogate.

The language of a right is warranted here because the interest at stake concerns not only whether simulation produces undesirable consequences but also whether the represented person has the normative standing to authorize or refuse a particular use of their identity. Therefore, the proposed right is grounded primarily in autonomy and authority rather than in ownership of identity or demonstrable downstream harm. Kirkpatrick’s account of algorithmic conscription is useful here because it describes an individual’s authority over whether their identity may be used as a generative resource for synthetic performance (Kirkpatrick, 2026). However, interactive simulation raises the further question of whether such a synthetic presence may be made available to others for social interaction.

Relational accounts of autonomy likewise resist treating autonomy as merely a matter of isolated individual choice, emphasizing the social and interpersonal conditions through which agency is exercised (Mackenzie and Stoljar, 2000; Nedelsky, 2012; Westlund, 2009). This relational basis also has precedent in privacy theory. Rachels (1975) argues that privacy matters, in part, because the ability to regulate access to ourselves enables different kinds of social relationships to be maintained. Person-specific simulation complicates this capacity by allowing another party to create a form of person-specific interaction without the represented individual determining the terms on which that interaction becomes available. Danaher and Nyholm (2025) likewise treat informed and ongoing consent as relevant to the permissible creation and use of personalized digital duplicates. Together, these approaches support an interest in determining not simply what information others possess or what representations they create, but whether one’s identity may be instantiated as a socially responsive surrogate through which others can conduct person-specific interactions.

As Section 3 showed, existing accounts of privacy, synthetic likeness, digital humans, and representational autonomy explain important aspects of the problem. The Right Not to Be Simulated addresses the narrower normative question raised by relational access: whether another party should presumptively be entitled to create or use an interactive surrogate associated with an identifiable person without that person’s authorization.

The possibility of a wrong without demonstrable downstream harm also has precedent in work on synthetic media. de Ruiter (2021) argues that the wrong of deepfakes cannot be reduced to consequences such as deception, reputational damage, or other harms that may follow from their circulation. Although person-specific interactive simulation is not equivalent to a deepfake, the distinction is relevant here: the normative objection may concern what has been done with a person’s identity, even when the resulting artifact produces no further measurable harm.

The autonomy basis of the claim becomes clearer in a limiting case. Suppose someone creates a highly person-specific simulation of another individual, interacts with it once in private, immediately deletes it, never distributes or commercializes it, and the represented person never discovers that it existed. In that case, there is little basis for claiming substantial consequential harm to the represented person. Nevertheless, the absence of downstream harm does not necessarily eliminate the wrong. If the person’s identity has been used without authorization to create an interactive surrogate, an autonomy or authority interest may already have been infringed, even if the resulting infringement is comparatively weak. Therefore, the claim is not simply a right against harmful consequences of simulation. It concerns whether another party was entitled to instantiate and interact with the person-specific surrogate in the first place.

This does not make the private, ephemeral case normatively equivalent to a persistent replica that is publicly distributed, commercially exploited, or used to deceive others. The former may constitute only a comparatively weak infringement of the presumptive claim, whereas the latter can implicate additional interests and cause substantial harms. Therefore, persistence, dissemination, commercial use, power asymmetry, vulnerability, and effects on real relationships can increase the seriousness of an unauthorized simulation without being necessary conditions for the underlying autonomy concern to arise.

This limiting case also clarifies the distinction between relational access and its possible consequences. Relational access does not require a persistent relationship or an accumulated interactional history. Rather, it arises when another person’s identity is instantiated as a sufficiently person-specific, socially responsive surrogate available for interaction. Repetition, memory, persistence, circulation, and effects on subsequent relationships can intensify the resulting concern, but they are not what first creates it. The underlying autonomy question is whether the user was entitled to make another person’s identity socially operative in this way at all. In an ephemeral private case that claim may be very weak and readily outweighed by competing interests, but it is not therefore identical to imagination: the imagined person has not been instantiated as an external responsive surrogate with which the user can interact.

The proposed right also extends beyond representational autonomy. Representational autonomy concerns how a person may be presented through identity-linked representations; the **Right Not to Be Simulated** would also address whether such a representation may be made socially operative as an apparent actor. The issue is not only what may be shown or said about a person, but also what a computational surrogate may be made to do in association with that person.

Contextual integrity provides a further basis for the right. Information disclosed or legitimately available in one context does not thereby become appropriate for every subsequent use (Nissenbaum, 2004; 2010). Photographs, recordings, professional interactions, or social media posts may all contribute to a convincing simulation, yet their availability does not imply authorization to convert them into an interactive surrogate. The availability of information about a person does not entail the person’s availability. More precisely, it does not entail authorization to transform that information into an interactive surrogate associated with the person.

### 4.1 Limits and competing interests

A presumptive right must also have limits. The clearest starting point is private imagination. People routinely imagine conversations, relationships, sexual encounters, conflicts, or alternative versions of other people without seeking their permission. A Right Not to Be Simulated cannot plausibly amount to a right against being imagined. The harder question is whether externalizing such imagination through computation changes its normative character. A person-specific simulation does something that imagination alone does not: it instantiates another person’s identity as an external, socially responsive artifact with which the user can interact. Therefore, the distinction between imagination and simulation is not that one necessarily causes harm while the other does not. Computational externalization makes another person’s identity operative as an interactive artifact outside the user’s mind. That artifact may also be retained, copied, repeatedly interacted with, modified, or circulated, but these possibilities increase the seriousness of the concern rather than constituting its sole basis.

A closely related problem arises in Öhman’s “pervert’s dilemma,” which asks why non-consensual deepfake pornography should be morally objectionable if private sexual fantasies involving another person are ordinarily permissible without that person’s consent (Öhman, 2020). Öhman argues that the apparent contradiction cannot be resolved by treating deepfakes and fantasies at a high level of abstraction; their moral significance also depends on the wider social context in which they occur. The dilemma is useful here, even though person-specific simulation extends beyond sexual deepfakes. “Hollow Pursuits” poses a similar boundary problem: if Barclay is permitted to imagine his colleagues privately, what changes when those fantasies are externalized as interactive simulations of identifiable people? Computational externalization does not make every fantasy impermissible, but the instantiation of an identifiable person as a responsive surrogate helps explain why simulation can give rise to a presumptive claim where private imagination does not, and why that claim may vary substantially in strength.

Other cases involve competing expressive interests. Biopics, historical fiction, parody, satire, and artistic representations routinely depict identifiable people without treating consent as an absolute requirement. Public figures, in particular, may legitimately be represented, criticized, imitated, or satirized. The proposed right should not collapse these practices into impermissible simulation merely because computational techniques are involved. Relevant considerations include whether a representation is presented as fiction or commentary, whether person-specific interaction is central to its function, whether generated conduct is attributed to the represented individual, and whether restricting the simulation would unduly interfere with legitimate expression. These competing interests support treating the right as presumptive rather than absolute.

There may also be socially beneficial uses of person-specific simulation. Medical training, education, research, accessibility applications, and therapeutic role-play may provide legitimate reasons for creating interactive representations without making every instance permissible. In some cases, a simulation may be sufficiently fictionalized or generalized that it no longer meaningfully represents a particular person. In others, a strong therapeutic, educational, or public interest may justify its use, subject to appropriate safeguards. Therefore, the proposed right creates a presumption that must be justified, not an automatic veto over every computational model that draws on information associated with an individual.

Simulations of deceased persons present a related but distinct boundary case. Research on the digital afterlife has already examined the ethical questions that arise when digital remains are used to represent the dead or to computationally reconstitute them (Öhman and Floridi, 2017; 2018). So-called deathbots or grief bots extend this problem by enabling interactive encounters with computational representations of deceased persons (Lindemann, 2022; Jiménez-Alonso and Brescó de Luna, 2023). In these cases, the represented person can no longer exercise autonomy or provide contemporaneous consent, while the simulation may serve memorial, therapeutic, commercial, or other purposes. Such cases raise additional questions about prior wishes, posthumous identity, the interests of surviving relatives, and the responsibilities of those who create or provide the simulation (Hollanek and Nowaczyk-Basińska, 2024). They do not map straightforwardly onto a right grounded in the present autonomy of a living person, but they demonstrate that person-specific simulation cannot be governed solely by contemporaneous consent.

### 4.2 Factors affecting the strength of the claim

Because the Right Not to Be Simulated is presumptive rather than absolute, its force varies across cases. Recent work on personalized digital duplicates likewise identifies consent, transparency, potential harms, and the context of use as relevant to permissibility (Danaher and Nyholm, 2025). These considerations are not intended as a validated or exhaustive framework but rather as factors that may strengthen or weaken the presumptive claim across different forms of simulation. Authorization will often provide the clearest justification for person-specific simulation, but it should not be treated as unlimited or irrevocable permission for every subsequent use. Its significance depends on what was authorized, for what purpose, for how long, and with what possibilities for withdrawal or reuse.

Person-specificity and attribution concern how strongly the simulation is tied to an identifiable individual. A generic character inspired by broad traits makes a weaker claim than a surrogate presented as a particular person. Relevant considerations include likeness, voice, biographical information, distinctive behavior, naming, and the likelihood that the generated conduct will be attributed to the represented individual. Treating these considerations together also avoids presenting identifiability and attribution as wholly independent, since they frequently overlap.

Persistence, distribution, and consequences concern what happens to the surrogate and the interactions conducted through it. An ephemeral private simulation differs from one that is retained, shared, searchable, commercially exploited, or made available at scale. The seriousness of the claim may increase further when the simulation affects reputation, employment, safety, intimate relationships, or the represented person’s ability to shape their social identity. Vulnerability may amplify these concerns even when the simulation’s technical characteristics are otherwise similar.

These considerations do not mechanically determine the permissibility of a simulation, nor do they establish a threshold beyond which the Right Not to Be Simulated automatically overrides competing interests. Instead, they clarify why the presumptive claim can vary considerably in strength. A private, ephemeral, and weakly person-specific simulation may present a comparatively weak claim, whereas a persistent, highly identifiable, interactive surrogate deployed commercially or within a substantial power asymmetry may present a much stronger one. The normative question remains whether the reasons for creating or using the simulation are sufficient to overcome the represented person’s presumptive claim against unauthorized instantiation and relational use.

The *Star Trek* cases illustrate how these considerations can interact. Barclay’s simulations are private and apparently limited in distribution, which weakens some consequential concerns without necessarily eliminating the underlying autonomy or authority claim. They are highly person-specific, interactive, and situated within an existing network of workplace relationships. The Brahms simulation begins with a legitimate professional purpose but develops into a person-specific encounter, and the behavior it generates becomes entangled with La Forge’s expectations of the real Brahms. Neither case is objectionable simply because a recognizable representation exists. What matters is the combination of person-specificity, interactive capacity, context, purpose, persistence, power, and the uses to which the surrogate is put. The Right Not to Be Simulated names the presumptive claim that such uses of person-specific identity require justification rather than being assumed permissible merely because the information or technical capacity needed to create them is available.

## 5 What Star Trek gets right (and wrong)

What *Star Trek* gets right is the discomfort that arises when a person-specific interactive surrogate becomes available for another person’s use. In “Hollow Pursuits,” the boundary is immediately legible, though not clearly articulated: the crew recognizes Barclay’s simulations as troubling, yet the episode frames the problem largely through workplace conduct, intimacy, and acceptable Holodeck use rather than asking whether the represented individuals have a claim to being simulated at all. “Booby Trap” and “Galaxy’s Child” expose the relational consequences more directly. La Forge’s interaction with the simulated Brahms creates expectations that shape his encounter with the real person. Contemporary HCI research reports closely related concerns: participants considering AI-clone scenarios anticipated that interactions with a clone could create false impressions of the source individual, alter subsequent interpersonal relationships, and cause users to attribute clone-generated experiences to the person represented (Lee et al., 2023). Figure 2 summarizes the different aspects of person-specific simulation made visible by the three episodes: the transformation of professional information into an interactive surrogate in “Booby Trap,” private person-specific simulation in “Hollow Pursuits,” and the consequences of an accumulated interactional history for the represented person in “Galaxy’s Child.”

**FIGURE 2:**
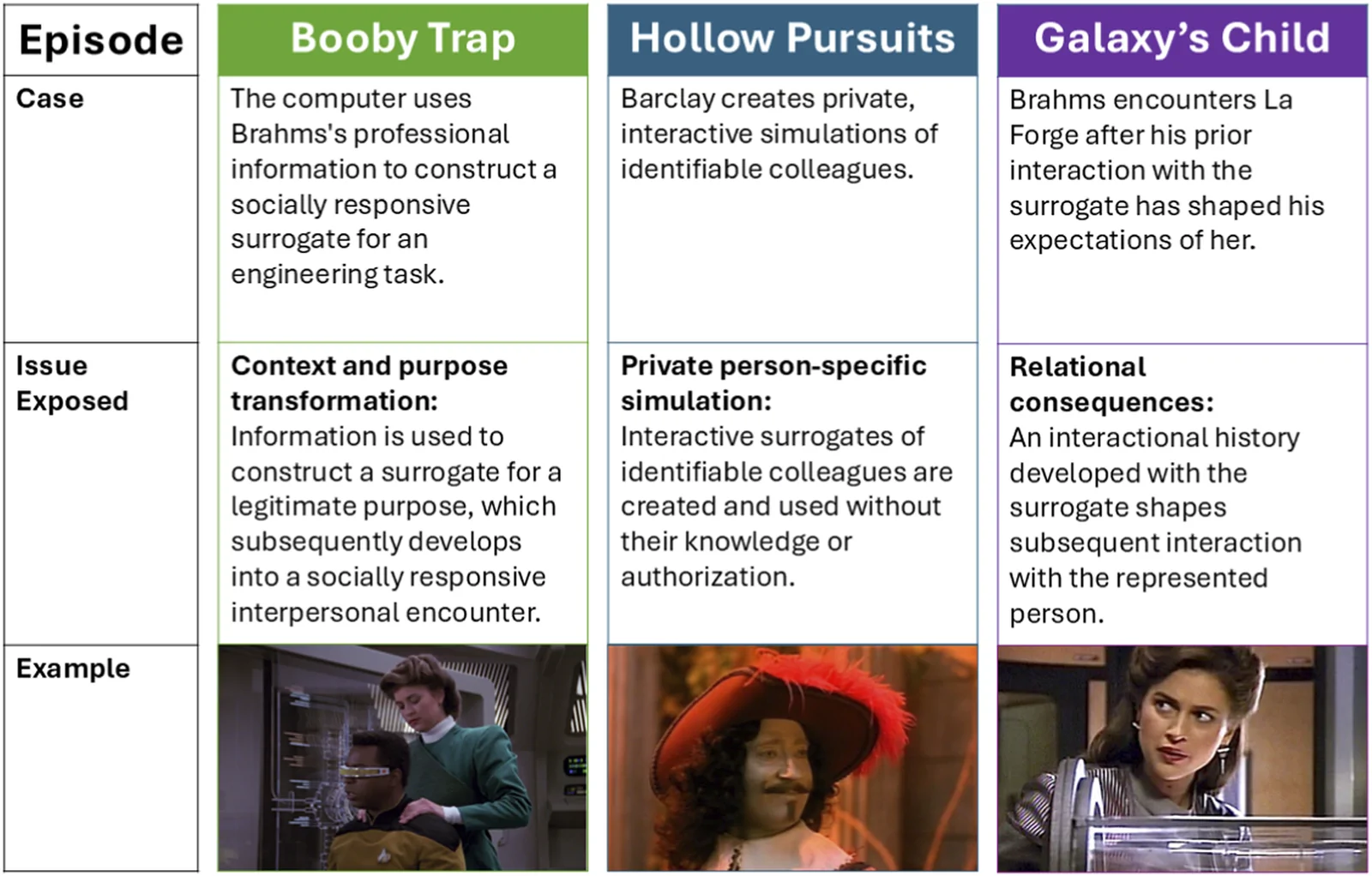
Three conceptual cases illustrating different aspects of person-specific simulation. The cases are presented comparatively rather than as stages in a necessary sequence.

Where *Star Trek* falls short is in treating these problems primarily as matters of individual conduct after a simulation has already been created. Barclay and La Forge become the focus of interpersonal conflict, while the technological infrastructure that enables person-specific simulation remains largely unquestioned. The Holodeck appears to impose few meaningful constraints on creating identifiable surrogates: Barclay can construct and modify interactive versions of his colleagues, while the *Enterprise* computer can transform professional information about Leah Brahms into a socially responsive model that evolves beyond its initial engineering purpose. In neither case does the system appear to distinguish between having access to information associated with a person and having authority to use that information to instantiate an interactive surrogate.

As previous work using video games to examine AI ethics and data politics has argued, fictional and interactive media can provide spaces in which emerging technological relationships are made available for ethical examination (Young et al., 2025). From the perspective of anticipatory governance, this omission from *Star Trek* is particularly instructive. Anticipatory governance is concerned not with predicting a single technological future but with developing capacities for foresight, engagement, and reflection while emerging technological trajectories remain open to intervention (Guston, 2014). Read this way, the Holodeck cases raise a governance question before its institutional settlement: technical access to person-specific information does not, by itself, determine whether that information should be used to create an interactive surrogate. A system responsive to the distinction developed here would therefore need to treat person-specific simulation as a use requiring justification, rather than assuming that existing access to the necessary data is sufficient authorization.

### 5.1 Existing law and the residual governance question

Existing law already addresses key aspects of this problem. In the European Union, the GDPR regulates subsequent processing of personal data and requires purpose limitation (European Parliament and Council of the European Union, 2016, Art. 5 (1)(b)). The transformation of photographs, voice recordings, biometric data, professional records, or other personal information into a person-specific simulation may therefore raise questions about lawful processing, purpose limitation, transparency, and data-subject rights. Article 50 of the AI Act addresses a different aspect of the problem through transparency requirements relevant to AI interaction and synthetic or manipulated content (European Parliament and Council of the European Union, 2024, Art. 50). Such transparency, however, does not determine whether an identifiable person’s identity may permissibly be used as the basis for that interaction.

More targeted protections for digital replicas have also emerged in the United States. These measures build in part on existing rights of publicity and personality, which protect interests in identity-linked features such as name, image, likeness, and voice, particularly against specified forms of unauthorized exploitation. Tennessee’s Ensuring Likeness, Voice, and Image Security (ELVIS) Act extends the state’s existing protection of name, photograph, and likeness to include voice, including readily identifiable simulations of an individual’s voice, while retaining exceptions for uses such as criticism, scholarship, satire, and parody (Tennessee General Assembly, 2024). California regulates certain contractual uses of digital replicas and unauthorized replicas of deceased personalities (California Legislature, 2024a; California Legislature, 2024b). The proposed federal NO FAKES Act of 2026, reported by the Senate Judiciary Committee in June 2026, would establish rights concerning highly realistic computer-generated replicas of an individual’s voice or visual likeness (United States Congress, 2026).

Denmark has likewise developed draft amendments to its Copyright Act that would protect against the public availability of realistic digitally generated imitations of personal characteristics without consent; the proposal subsequently entered the EU technical-regulation notification process (Kulturministeriet, 2025). These measures demonstrate that person-specific synthetic media are increasingly subject to legal regulation, but they address different interests and stages of the process: data processing, transparency, contractual authorization, likeness, public dissemination, and commercial or expressive use (Bosher, 2026). They therefore do not render the normative question posed here redundant: whether the creation or use of an interactive person-specific surrogate itself gives rise to a presumptive claim on the part of the person represented.

The Right Not to Be Simulated is therefore not proposed on the assumption that existing law is silent on person-specific simulation. Existing regimes may regulate the data used to construct a surrogate, the appropriation of protected features, deceptive or harmful outputs, commercial exploitation, and transparency obligations. The residual normative question is narrower: whether an identifiable person has a presumptive claim against having their identity instantiated as an interactive surrogate, even when no single existing doctrine clearly prohibits the resulting simulation. The proposed right provides a principle for assessing existing and future governance mechanisms, rather than a claim that they must all be replaced by a new freestanding legal right.

### 5.2 From normative claim to governance responsibilities

Treating the Right Not to Be Simulated as a normative principle also helps distinguish responsibilities that arise at different stages of a surrogate’s creation and use. Users and creators are responsible for determining whether constructing or interacting with a person-specific simulation is justified, particularly when they knowingly use another person’s identity without authorization. Platforms and service providers can determine whether person-specific simulations are permitted, what forms of authorization are required, whether represented individuals can report or contest them, and whether simulations can be retained, shared, or made publicly accessible. Developers make upstream design choices about whether systems facilitate person-specific imitation, what safeguards are technically available, and whether provenance, authorization, deletion, or identity-protection mechanisms are built into the system. Therefore, the existence of user responsibility does not exhaust the responsibilities of the infrastructure through which relational access becomes possible. The Brahms case illustrates this mixed agency: La Forge actively shapes the encounter, but the Holodeck infrastructure determines what forms of person-specific generative behavior are available to him.

General-purpose generative AI complicates the allocation of responsibility because a person-specific surrogate need not be a persistent, standalone avatar or dedicated program. A user may prompt a general-purpose language model to respond as an identifiable living person, producing an ephemeral simulation generated anew within a conversation. On the account developed here, persistence is not a necessary condition for relational access: what matters is whether the system produces a sufficiently person-specific and socially responsive surrogate capable of supporting the form of interaction defined here as relational access. At the same time, the ephemeral character of such a simulation may weaken the presumptive claim relative to a persistent model that is retained, distributed, commercialized, or made available to many users. This distinction reinforces the need for governance mechanisms sensitive to both the capabilities of general-purpose systems and the contexts in which users invoke them.

Possible governance responses need not begin with prohibition. Research on AI clones has similarly identified active consent and ongoing source-person control as key design considerations (Lee et al., 2023). Possible mechanisms could include scoped authorization for simulation, opportunities to audit or contest unauthorized surrogates, restrictions on persistence or redistribution, and a clear separation between professional or informational access to personal data and permission to use those data for person-specific interaction.

The Right Not to Be Simulated offers a way to name the principle that these stories approach without explicitly articulating it. Its purpose is not to treat every unauthorized simulation as equally serious, nor to assume that a new legal prohibition must attach to every infringement of the normative claim. Rather, it shifts attention to a question that should arise before enabling person-specific interaction: what, if anything, justifies using an identifiable person’s identity as the basis for an interactive surrogate? In this sense, the Holodeck’s continuing relevance lies not simply in its technological prescience but in showing how easily the technical capacity to simulate another person can precede the social, institutional, and legal arrangements needed to govern that capacity.

## 6 Conclusion

The Holodeck’s enduring relevance to contemporary debates about XR and AI lies not only in anticipating immersive virtual environments but also in depicting what happens when information associated with a person becomes operational as an interactive surrogate. Across “Booby Trap,” “Hollow Pursuits,” and “Galaxy’s Child,” *Star Trek: The Next-Generation* shows how person-specific information can be transformed into a socially responsive model, used without the represented person’s participation, and produce interactional histories that affect subsequent relationships. The ethical concern therefore extends beyond whether personal information has been appropriately collected or processed, whether a likeness is accurate, or whether generated behavior accurately represents the person.

I describe this possibility as relational access. Whereas surveillance provides epistemic access and profiling provides inferential access, person-specific simulation can provide access to a socially responsive surrogate through which users conduct person-specific interactions. Repeated encounters may produce an accumulated interactional history, but such a history is a possible consequence of relational access rather than a necessary condition for it.

The proposed Right Not to Be Simulated articulates the corresponding normative claim. It is grounded primarily in autonomy and authority rather than in ownership of identity or a requirement to demonstrate downstream harm. Even a private, ephemeral simulation that is never distributed or discovered may raise the question of whether another party was entitled to instantiate an identifiable person as an interactive surrogate in the first place. The claim is presumptive rather than absolute, and its strength varies with person-specificity and attribution; interactivity, fidelity, and relational capacity; context, purpose, and power; and persistence, distribution, and consequences. Consent, legitimate expressive interests, professional or therapeutic purposes, and other competing considerations may justify particular cases. Therefore, the Right Not to Be Simulated requires consideration and justification rather than imposing a universal prohibition on person-specific representation or simulation.

Contemporary AI and XR systems remain far from the fictional capabilities of the Holodeck, yet the governance problem identified by *Star Trek: The Next-Generation* is already becoming visible. Digital systems increasingly generate speech, behavior, appearance, and interaction associated with identifiable individuals, while existing data-protection, likeness, digital-replica, and synthetic-media regimes regulate different parts of that process. The Right Not to Be Simulated is not intended to displace these protections or to assume that every infringement of the normative claim requires a new legal prohibition. Rather, it identifies a residual question that should inform the choices of users, developers, platforms, and policymakers: under what conditions, and with what justification, should an identifiable person’s identity be used as the basis for an interactive surrogate? *Star Trek*’s value as anticipatory governance lies in making that question visible before the technological capacity to create such surrogates becomes unremarkable.

## Data Availability

This article presents a theoretical and conceptual analysis. No new empirical datasets were generated or analysed in this study. Inquiries can be directed to the corresponding author.
